# Transcriptome profiling in engrailed-2 mutant mice reveals common molecular pathways associated with autism spectrum disorders

**DOI:** 10.1186/2040-2392-4-51

**Published:** 2013-12-19

**Authors:** Paola Sgadò, Giovanni Provenzano, Erik Dassi, Valentina Adami, Giulia Zunino, Sacha Genovesi, Simona Casarosa, Yuri Bozzi

**Affiliations:** 1 Laboratory of Molecular Neuropathology, Centre for Integrative Biology (CIBIO), University of Trento, Via delle Regole 101, 38123 Trento, Italy; 2 Laboratory of Translational Genomics, Centre for Integrative Biology (CIBIO), University of Trento, Via delle Regole 101, 38123 Trento, Italy; 3 High Throughput Screening Core Facility, Centre for Integrative Biology (CIBIO), University of Trento, Via delle Regole 101, 38123 Trento, Italy; 4 Laboratory of Developmental Neurobiology, Centre for Integrative Biology (CIBIO), University of Trento, Via delle Regole 101, 38123 Trento, Italy; 5 C.N.R. Neuroscience Institute, via G. Moruzzi 1, 56124 Pisa, Italy

**Keywords:** *En2*, Neurodevelopmental disorders, Mouse models, Immune response, Synaptic function, *Scn1a*, *Grm5*, *Nrxn3*

## Abstract

**Background:**

Transcriptome analysis has been used in autism spectrum disorder (ASD) to unravel common pathogenic pathways based on the assumption that distinct rare genetic variants or epigenetic modifications affect common biological pathways. To unravel recurrent ASD-related neuropathological mechanisms, we took advantage of the *En2*^*-/-*^ mouse model and performed transcriptome profiling on cerebellar and hippocampal adult tissues.

**Methods:**

Cerebellar and hippocampal tissue samples from three *En2*^*-/-*^ and wild type (WT) littermate mice were assessed for differential gene expression using microarray hybridization followed by RankProd analysis. To identify functional categories overrepresented in the differentially expressed genes, we used integrated gene-network analysis, gene ontology enrichment and mouse phenotype ontology analysis. Furthermore, we performed direct enrichment analysis of ASD-associated genes from the SFARI repository in our differentially expressed genes.

**Results:**

Given the limited number of animals used in the study, we used permissive criteria and identified 842 differentially expressed genes in *En2*^*-/-*^ cerebellum and 862 in the *En2*^*-/-*^ hippocampus. Our functional analysis revealed that the molecular signature of *En2*^*-/-*^ cerebellum and hippocampus shares convergent pathological pathways with ASD, including abnormal synaptic transmission, altered developmental processes and increased immune response. Furthermore, when directly compared to the repository of the SFARI database, our differentially expressed genes in the hippocampus showed enrichment of ASD-associated genes significantly higher than previously reported. qPCR was performed for representative genes to confirm relative transcript levels compared to those detected in microarrays.

**Conclusions:**

Despite the limited number of animals used in the study, our bioinformatic analysis indicates the *En2*^*-/-*^ mouse is a valuable tool for investigating molecular alterations related to ASD.

## Background

Autism spectrum disorder (ASD) defines a complex group of neurodevelopmental disabilities characterized by a wide range of impairments in social and communicative skills, stereotyped behaviors, and restricted mental flexibility [1]. The neurodevelopmental and neuroanatomical bases of ASD have been confirmed by a number of clinical, neuroimaging and neuropathological studies [1-3]. The most evident abnormality in ASD consists in an early (perinatal) brain overgrowth followed by an arrest of growth during the first year of age [4]. Neuropathological studies on post-mortem samples from ASD patients also showed a number of cellular and cytoarchitectural abnormalities at the level of the cerebral cortex, cerebellum, amygdala and forebrain limbic structures. Anomalies in the cerebellum are the most reproducible neuropathological alterations in ASD patients [3,5].

A large series of evidence clearly indicates that neuropathological features and behavioral deficits of ASD have a primarily genetic origin. However, the etiology of ASD remains essentially unknown [6,7]. Transcriptome analysis has also been used to unravel common pathogenic pathways based on the assumption that distinct rare genetic variants or epigenetic modifications affect common biological pathways dysregulated in ASD [6]. Several studies have analyzed genome-wide expression profiles of ASD patients using lymphoblastoid cell lines and blood samples, supporting upregulation of immune genes and downregulation of neurodevelopmental genes as key players in the pathogenesis of ASD (see [8] for a review). Recently, gene co-expression network analysis of autistic brain areas revealed defects in cortical patterning and an enrichment of differentially expressed genes associated with ASD [9].

The homeobox-containing transcription factor *engrailed-2* (*En2*) is crucially involved in the regionalization, patterning and neuronal differentiation of the midbrain and hindbrain [10-15]. Human studies indicated association of two intronic single-nucleotide polymorphisms (SNPs) in the human *engrailed-2* (*EN2*) gene with ASD [16,17]. Furthermore, the ASD associated A-C haplotype markedly affected *EN2* promoter activity when tested with a luciferase reporter assay in rat, mouse and human cell lines [18]. A recent study of the epigenetic evaluation of *EN2* in post-mortem cerebellar samples from autistic patients indicated a persistent upregulation of this homeobox gene induced by epigenetic abnormalities in histone methylation patterns that may contribute to Purkinje cell loss in some individuals with autism [19].

Mice lacking the homeobox domain of *En2* (*En2*^*hd/hd*^ mice; [20], referred to as *En2*^*-/-*^) have been proposed as a model for ASD, due to their complex neuroanatomical and behavioral phenotype. *En2*^*-/-*^ mice display cerebellar hypoplasia, including a reduced number of Purkinje cells, and a defect in the antero-posterior pattern of cerebellar foliation [20-23]. The behavior of *En2*^*-/-*^ mice is also reminiscent of some features of ASD individuals. Deficits in social behaviors were detected in *En2*^*-/-*^ mice, including decreased play and reduced social interactions; locomotor impairment, as well as defective spatial learning and memory, was also reported in these mice [24-26]. Furthermore, we reported dysfunctions in GABAergic interneurons in adult *En2*^*-/-*^ mice and demonstrated engrailed protein expression in specific subpopulations of adult hippocampal and cortical interneurons [27].

To unravel recurrent ASD-related neuropathological mechanisms, we took advantage of the *En2*^*-/-*^ mouse model and performed genome-wide expression profiling on cerebellar and hippocampal adult tissues. Our transcriptome analysis of the cerebellum and hippocampus of *En2*^*-/-*^ mice suggests convergent pathological pathways with ASD, including abnormal synaptic transmission and increased immune response. Furthermore, we provide evidence for a significant enrichment of differentially expressed genes associated to ASD in this mouse model of the disease.

## Methods

### Animals

Experiments were conducted in conformity with the European Communities Council Directive of 24 November 1986 (86/609/EEC) and were approved by the Italian Ministry of Health and Ethics Committee of the University of Trento. Animals were housed in a 12 hr light/dark cycle with food and water available *ad libitum*. All surgery was performed under chloral hydrate anesthesia, and all efforts were made to minimize suffering. The generation of *En2*^*-/-*^ mice was previously described [20]. The original *En2* mutants (mixed 129Sv x C57BL/6 and outbred genetic background) were crossed at least five times into a C57BL/6 background. Heterozygous matings (*En2*^*+/-*^ x *En2*^*+/-*^) were used to generate the *En2*^*+/+*^ (wild type, WT) and *En2*^*-/-*^ littermates used in this study. PCR genotyping was performed according to the protocol available at the Jackson Laboratory website (http://www.jax.org; mouse strain *En2*^*tm1Alj*^). WT and *En2*^*-/-*^ age-matched adult (3 to 5 months old; weight = 25 to 35 g) littermates mice of both sexes were used.

### Microarrays

RNAs from dissected hippocampi and cerebella from three adult mice for each genotype were purified using standard column purification according to the manufacturer’s protocol (RNAeasy Mini Kit, Qiagen, USA). RNA quality was analyzed by microfluidic gel electrophoresis on RNA 6000 NanoChips using the Agilent 2100 Bioanalyzer. Only RNA with a high (>9) RNA integrity number was selected and used for subsequent retro-transcription, labeling and array hybridization according to Agilent protocols. Mouse gene expression arrays (Agilent 4X44K slides) were hybridized and scanned with the Agilent microarray station.

### Bioinformatics

Intensity values were processed with Agi4x44PreProcess (http://bioconductor.org/packages/2.12/bioc/html/Agi4x44PreProcess.html) using default parameters to remove low-quality probes. Signals were then normalized by means of the quantile normalization method. To evaluate differential expression, we used RankProd (http://www.bioconductor.org/packages/2.11/bioc/html/RankProd.html) [28]. RankProd utilizes the Rank Product (RP) non-parametric method [29] to identify up- or downregulated genes. The RP is equivalent to calculating the geometric mean rank with a statistical method (average rank) that is slightly more sensitive to outlier data and puts a higher premium on consistency between the ranks in various lists. To assess for functional categories overrepresented in the differentially expressed genes, we used DAVID (http://david.abcc.ncifcrf.gov) and Ingenuity Pathway Analysis (Ingenuity Systems, Inc., USA). To focus the functional analysis on brain expressed genes we used, as background for our functional analyses, a list of tissue specific ‘expressed genes’ for both the cerebellum and the hippocampus. Our ‘expressed genes’ lists were obtained by filtering the genes by the normalized expression values and excluding the ones with the lowest expression levels (<10th percentile), and include 13,652 genes for the cerebellum and 13,141 for the hippocampus. The hypergeometric test and the Student’s *t*-test were computed with R (http://www.r-project.org).

### Quantitative PCR

Total RNAs were extracted by Trizol™ reagent (Invitrogen Life Technologies, USA) from dissected hippocampi and cerebella from four WT and four *En2*^*-/-*^ adult mice. RNAs were DNAse-treated and purified with RNeasy Mini Kit (Qiagen, USA). cDNA was synthesized from pooled RNAs (2 μg) using the SuperScript™ VILO™ (Invitrogen Life Technologies, USA) according to the manufacturer’s instructions. Individual PCR reactions were conducted in a volume of 20 μl using the KAPA FAST SYBR qPCR kit (KAPABiosystems, USA) according to manufacturer’s instructions. Mouse mitochondrial ribosomal protein L41 (*Mrpl41*) was used as a standard for quantification as previously shown [30]. Primers (MWG, Germany) were designed on different exons to avoid amplification of genomic DNA. A list of primer sequences is reported in Additional file 1. Each PCR cycle consisted of denaturation for 10 s at 94°C, annealing for 20 s at 60°C and extension for 30 s at 72°C. The fluorescence intensity of SYBR green I was read and acquired at 72°C after completion of the extension step of each cycle. PCR conditions for individual primer sets were optimized by varying template cDNA and primer concentration in order to obtain a single PCR product and amplification efficiency >90%. Relative expression values were calculated using the Pfaffl method [31].

## Results

### Differential gene expression in cerebellum and hippocampus of En2^-/-^ mice

**Figure 1:**
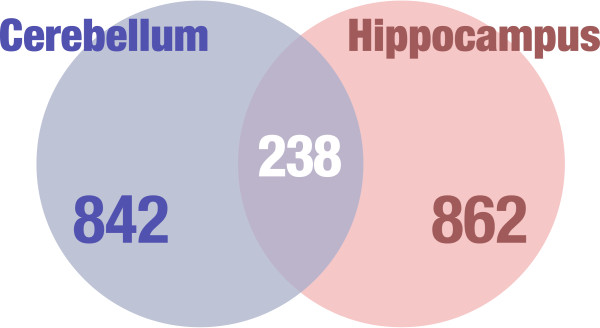
**Venn diagram of differentially expressed genes in *En2***^***-/-***^**adult cerebellum and hippocampus.** A total of 842 and 862 differentially expressed genes were identified in the adult cerebellum (CB, blue) and hippocampus (Hippo, red) of *En2*^*-/-*^ mice, respectively. Among these, 238 show differential expression in both tissues.

The cerebellum of *En2*^*-/-*^ mice shows Purkinje cell loss and structural abnormalities resembling the neuropathological features observed in ASD patients [20,21,23]. To identify genes and pathways altered in *En2*^*-/-*^ mice, we initially performed transcriptome profiling of the whole cerebellar tissue. *En2*^*-/-*^ and WT cerebella from adult mice were assessed for differential gene expression by microarray and bioinformatical analysis (see Methods). We found 842 differentially expressed genes in the cerebellum of *En2*^*-/-*^ mice compared to their control littermates. Among these, 407 and 435 were up- and downregulated, respectively. Alterations in limbic structures have also been shown in the temporal lobes of autistic patients. The main abnormalities were shown in the superior temporal sulcus and the ventral-basal temporal region, both of which are involved in decoding social stimuli and therefore are associated with the social deficits [32-34]. Most importantly, we previously showed anatomical defects in the *En2*^*-/-*^ hippocampus that might contribute to the behavioral deficits displayed by these mutants [27]. We therefore combined the cerebellar gene expression profile to that of the hippocampus. We found 862 differentially expressed genes in the hippocampus, among those 378 were upregulated and 484 were downregulated in *En2*^*-/-*^ mice compared to their littermate controls. Additional file 2 shows the entire list of genes differentially expressed in the cerebellum and hippocampus of *En2*^*-/-*^ mice with the differential expression *P* value and the percentage of false prediction (pfp) value calculated by RankProd. Given the profound structural and cytoarchitectural phenotype of the *En2*^*-/-*^ cerebellum and the variability of the phenotype among individuals, we chose to be more permissive and include genes with smaller differential expression fold changes applying a moderate cutoff to the uncorrected *P* value [see Additional file 2]. Differentially expressed genes, which are common in the hippocampus and the cerebellum, are summarized in Figure 1 and listed in Additional file 3. Remarkably, *En2* was not among the differentially expressed genes. The microarray probe for *En2* (A_51_P397876) was designed on the 3′ untranslated region of the gene that is not deleted in the *En2*^*-/-*^ locus [20]. Our data confirm the previous reports indicating the presence of a residual transcript from the *En2*^*-/-*^ locus [20]. Furthermore, real-time quantitative PCR (qPCR) analysis of En2 expression using homeobox specific primers revealed no expression of the full-length gene in *En2*^*-/-*^ mice [see Additional file 4].

### Functional analysis

**Figure 2:**
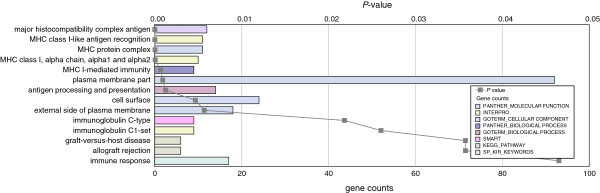
**Overrepresented gene ontology categories for differentially expressed genes in the *En2***^***-/-***^**adult cerebellum.** Differentially expressed genes (n = 842) were analyzed for enrichment in gene ontology categories using DAVID with a Benjamini corrected *P* value cutoff of 0.05. Categories are arranged from most significant and downwards (gray line), for each category the number of genes is indicated by the length of the horizontal bars (gene counts). To highlight distinct ontological categories, bars are color-coded as indicated in the inset to the figure. Data details are also reported in Additional file 5.

**Figure 3:**
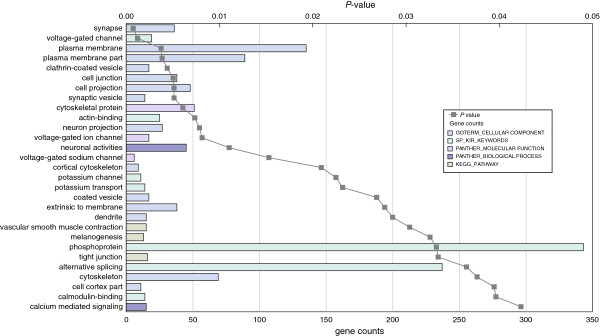
**Overrepresented gene ontology categories for differentially expressed genes in the *En2***^***-/-***^**adult hippocampus.** Differentially expressed genes (n = 862) were analyzed for enrichment in gene ontology categories using public databases with a Benjamini corrected *P* value cutoff of 0.05. Further details regarding the figure and inset are described in the legend to Figure 2. Data details are also reported in Additional file 5.

To explore the biological processes most relevant to the phenotype of the *En2*^*-/-*^ mice, the differentially expressed genes were analyzed through integrated gene-network analysis using the curated Ingenuity Pathway Analysis (IPA) database and the Database for Annotation, Visualization and Integrated Discovery (DAVID). Additional file 5 shows the most significantly enriched disease and cellular function categories obtained with IPA and gene ontology analysis. Functional categories included increased seizure and decreased neurotransmission release in the hippocampus, and decreased cancer-related diseases and development of lymphocytes as cellular function in the cerebellum. To identify statistically significant over-representation of key neurobiological processes, functional annotation analysis was performed with DAVID using a tissue specific list of ‘expressed genes’ as background (see Methods). To verify the tissue-expression pattern of the samples, we first classified the differentially expressed genes based on their tissue expression (*P* <0.05, calculated using Benjamini multiple testing correction). We only found significant tissue expression terms for *En2*^*-/-*^ hippocampus showing significant enrichment in expression of genes related to ‘brain cortex’, ‘brain’, ‘hypothalamus’, ‘eye’ and ‘hippocampus’ [see Additional file 6]. We then analyzed the functional annotation using gene sets from the gene ontology (GO) public databases and our ‘expressed genes’ list as background. Figure 2 shows all GO terms enriched in the differentially expressed genes of *En2*^*-/-*^ cerebellum with the gene counts and the relative *P* value (*P* <0.05, calculated using Benjamini multiple testing correction). Among the most represented functional categories were several terms related to the major histocompatibility complex (MHC)-mediated immunity and immune response. The GO terms related to the *En2*^*-/-*^ hippocampus are shown in Figure 3 with the gene counts and the relative *P* value (*P* <0.05, calculated using Benjamini multiple testing correction). Of the enriched terms, many related to the cellular components synapse, synaptic vesicle and neuronal projection. Within the biological processes, the most represented terms were ‘neuronal activities’ and ‘calcium mediated signal’. Interestingly, the keyword ‘phosphoprotein’ was significantly enriched, suggesting a generic alteration in the protein phosphorylation state in the *En2*^*-/-*^ hippocampus.

**Figure 4:**
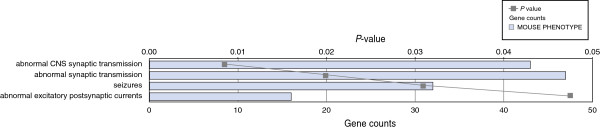
**Mouse phenotype categories associated with differentially expressed genes in the *En2***^***-/-***^**hippocampus.** Differentially expressed genes in *En2*^*-/-*^ hippocampus were analyzed for enrichment in mouse phenotypes using ToppGene with a corrected *P* value cutoff of 0.05. Categories are arranged from most significant and downwards (gray line); number of genes in the category is indicated by the length of the horizontal bars (gene counts).

To assess the functional consequences of *En2* ablation, we analyzed enrichment of the differentially expressed genes based on the mouse phenotype from the Mammalian Phenotype Ontology (MPO) project [35], using ToppGene [36]. In the *En2*^*-/-*^ cerebellum the only significantly enriched mouse phenotype was ‘loss of dopaminergic neurons’, a term associated with the cerebellar phenotype by the role of *En2* in midbrain/hindbrain development and dopaminergic neuron survival [37,38]. For the hippocampus we found significant enrichment for terms related to seizure and altered synaptic transmission (Figure 4). These data are in accordance with our previously described increase in seizure susceptibility and decrease in GABAergic neuron subpopulations in *En2*^*-/-*^ mice [27,30].

Remarkably, when analyzed specifically for ASD-associated genes, the differentially expressed genes showed significant over-representation of known ASD susceptibility genes when compared directly to the repository of the Simons Foundation and Autism Research Initiative (SFARI) database (gene.sfari.org). Since the SFARI database comprises genes related to neurodevelopmental disorders, therefore expressed in the brain, we filtered the SFARI database gene list with our list of ‘expressed genes’ in the cerebellum and in the hippocampus (see Methods for details). We used these SFARI gene lists to calculate enrichment with our differentially expressed genes. Table 1 shows the list of the ASD-associated differentially expressed genes for the cerebellum and the hippocampus. To compute significant enrichment between our differentially expressed genes and the SFARI genes, we employed the hypergeometric test. The statistical analysis indicated significant enrichment only for the hippocampus (*P* <0.05), whereas no significant enrichment was observed for the cerebellum.

**Table 1:** **Enrichment of autism spectrum disorder (ASD)-related genes in *En2***^***-/- ***^**cerebellum and hippocampus differentially expressed genes**

**(a) Cerebellum**			
**Gene Symbol**	**Gene Name**	***P *** **value**	**Fold change**
*Ada*	adenosine deaminase	3.50E-03	1.705
*Ahi1*	Abelson helper integration site 1	4.70E-03	1.690
*Cacna1g*	calcium channel, voltage-dependent, T type, alpha 1G subunit	8.20E-03	0.670
*Cdh10*	cadherin 10	4.40E-03	1.662
*Eml1*	echinoderm microtubule associated protein like 1	2.80E-03	0.581
*Erbb4*	v-erb-a erythroblastic leukemia viral oncogene homolog 4 (avian)	9.90E-03	0.653
*Glo1*	glyoxalase 1	6.00E-03	0.625
*Gnas*	GNAS (guanine nucleotide binding protein, alpha stimulating) complex locus	5.60E-03	1.565
*Grm5*	glutamate receptor, metabotropic 5	3.70E-03	1.656
*Itgb7*	integrin beta 7	1.00E-04	0.453
*Kdm5c*	lysine (K)-specific demethylase 5C	8.50E-03	0.660
*Kit*	kit oncogene	3.00E-03	0.591
*Nrp2*	neuropilin 2	0.00	2.503
*Nrxn3*	neurexin III	1.80E-03	1.843
*Park2*	Parkinson disease (autosomal recessive, juvenile) 2, parkin	5.20E-03	1.723
*Pinx1*	PIN2/TERF1 interacting, telomerase inhibitor 1	1.00E-03	1.871
*Plcb1*	phospholipase C, beta 1	3.60E-03	1.677
*Rb1cc1*	RB1-inducible coiled-coil 1	6.90E-03	1.635
*Rpp25*	ribonuclease P 25 subunit (human)	8.00E-03	0.619
*Stk39*	serine/threonine kinase 39, STE20/SPS1 homolog (yeast)	9.40E-03	1.548
*Th*	tyrosine hydroxylase	7.90E-03	1.564
**(b) Hippocampus**			
**Gene Symbol**	**Gene Name**	***P *** **value**	**Fold change**
*Aff4*	AF4/FMR2 family, member 4	1.30E-03	4.490
*Atp2b2*	ATPase, Ca++ transporting, plasma membrane 2	2.80E-03	0.544
*Baiap2*	brain-specific angiogenesis inhibitor 1-associated protein 2	8.00E-04	0.478
*Camta1*	calmodulin binding transcription activator 1	4.40E-03	0.561
*Dab1*	disabled homolog 1 (Drosophila)	4.10E-03	0.552
*Dctn5*	dynactin 5	4.10E-03	1.733
*Dlg4*	discs, large homolog 4 (Drosophila)	0.00	0.346
*Egr2*	early growth response 2	7.00E-04	2.099
*Eif4ebp2*	eukaryotic translation initiation factor 4E binding protein 2	3.90E-03	0.551
*Ep400*	E1A binding protein p400	2.00E-04	2.361
*Foxp1*	forkhead box P1	3.00E-04	0.425
*Gabra4*	gamma-aminobutyric acid (GABA) A receptor, subunit alpha 4	4.00E-04	2.151
*Gnas*	GNAS (guanine nucleotide binding protein, alpha stimulating) complex locus	3.90E-03	0.555
*Gsk3b*	glycogen synthase kinase 3 beta	3.50E-03	0.541
*Gtf2i*	general transcription factor II I	3.80E-03	0.552
*Kit*	kit oncogene	2.00E-04	0.417
*Klc2*	kinesin light chain 2	4.80E-03	0.565
*Lrrc1*	leucine rich repeat containing 1	4.20E-03	1.742
*Nrcam*	neuron-glia-CAM-related cell adhesion molecule	3.10E-03	1.777
*Ntng1*	netrin G1	1.90E-03	1.842
*Ntrk3*	neurotrophic tyrosine kinase, receptor, type 3	1.00E-04	0.426
*Park2*	Parkinson disease (autosomal recessive, juvenile) 2, parkin	1.00E-04	2.402
*Plcb1*	phospholipase C, beta 1	8.00E-04	0.487
*Prkcb*	protein kinase C, beta	8.00E-04	0.490
*Rpp25*	ribonuclease P 25 subunit (human)	1.10E-03	0.487
*Sbf1*	SET binding factor 1	4.40E-03	0.562
*Scn1a*	sodium channel, voltage-gated, type I, alpha	1.00E-04	0.410
*Scn8a*	sodium channel, voltage-gated, type VIII, alpha	1.00E-04	0.414
*Syn1*	synapsin I	5.00E-04	0.469
*Syne1*	synaptic nuclear envelope 1	4.80E-03	0.562
*Thra*	thyroid hormone receptor alpha	1.00E-04	0.419
*Ube2h*	ubiquitin-conjugating enzyme E2H	4.00E-03	0.559
*Ubl7*	ubiquitin-like 7 (bone marrow stromal cell-derived)	3.70E-03	0.557

ASD-related genes enriched in the *En2*^*-/-*^ (**a**) cerebellum and (**b**) hippocampus with their differential expression *P* value and fold change.

To compare our findings with the three major genome-wide expression studies on ASD brain tissue [9,39,40], we matched the publicly available differentially expressed genes with the same ASD-related gene lists that we used for our analysis. To increase accuracy, we computed, for each study, the hypergeometric test and obtained an enrichment *P* value that we used for direct comparison. Table 2 summarizes the results of the enrichment analyses, separated in tissue-specific groups, and the comparison between the studies. For Voineagu *et al.*[9] we re-analyzed the cerebellum data using the GEO2R tool with default parameters and used these results to evaluate the correspondence with our study. The results for cerebellum show a significant enrichment with ASD-associated genes only for the Voineagu *et al.* study (4.75% enrichment, *P* = 0.0343). Our study, however, was the only one to display significant enrichment of ASD-related genes (4.24% enrichment, p = 0.0265) in the limbic regions.

**Table 2:** Correlation of Simons Foundation and Autism Research Initiative (SFARI) database genes with published transcriptome studies in Autism Spectrum Disorder (ASD) brain and our study

**Cerebellum**	**# SFARI genes**	**% enrichment**	***P *** **value**	**Gene names**
**This study** (3 *En2*^*-/-*^, 3 WT)	21	2.79%	4.98E-01	*Ada,* ***Ahi1****,* ***Cacna1g****, Cdh10, Eml1, Erbb4, Glo1, Gnas, Grm5, Itgb7, Kdm5c, Kit, Nrp2, Nrxn3, Park2, Pinx1, Plcb1, Rb1cc1, Rpp25, Stk39, Th*
**Voineagu *et al.*** [9] (11 autism, 10 controls)	16	4.75%	3.43E-02 (*)	***AHI1****, ANK3,* ***CACNA1G****, CBS,* ***EN2****, EPHB6, FAT1, FOXP1,GAP43, GRIN2A, HSD11B1, NLGN3, NTNG1, RAB11FIP5, SLC30A5, UBE3A*
**Purcell *et al.*** [39] (9 autism, 9 controls)	1	3.33%	5.85E-01	*CNR1*
**Limbic regions**	**# SFARI genes**	**% enrichment**	***P *** **value**	**Gene names**
**This study** (3 *En2*^*-/-*^, 3 WT)	33	4.24%	2.65E-02 (*)	*Aff4,* ***Atp2b2****, Baiap2, Camta1, Dab1, Dctn5, Dlg4, Egr2,* ***Eif4ebp2****, Ep400, Foxp1, Gabra4, Gnas, Gsk3b, Gtf2i, Kit, Klc2, Lrrc1, Nrcam, Ntng1,* ***Ntrk3****, Park2, Plcb1,* ***Prkcb****,* ***Rpp25****, Sbf1, Scn1a, Scn8a, Syn1, Syne1, Thra,* ***Ube2h****, Ubl7*
**Voineagu *et al.*** [9] (13 autism, 13 controls)	36	3.70%	1.04E-01	***AHI1****, APBA2,* ***ATP2B2****, ATRNL1, AUTS2, BCL2, BTAF1, CADM1, CD99L2, DNM1L, DPP10,* ***EIF4EBP2****, FAT1, GRIN2A, ICA1, MAOA,* ***MSN****,* ***NTRK3****, PCDH9, PPFIA1,* ***PRKCB****, PTCHD1, RAB11FIP5, RGS7,* ***RPP25****, SLC16A3, SLC25A12,* ***SLC9A9,*** *STXBP1, SYT17, TOMM20, TSC2, TUBGCP5,* ***UBE2H****, UBR5, UPF3B*
**Garbett *et al.*** [40] (6 autism, 6 controls)	4	3.05%	5.52E-01	***AHI1****,* ***MSN****, SDC2,* ***SLC9A9***

Percentage of enrichment calculated on SFARI ASD-associated genes compared to the number of differentially expressed genes. Differentially expressed genes in Voineagu *et al.*[9] calculated using GEO2R analysis. Genes present in at least two of the studies are shown in bold. Enrichment *P* values calculated with the hypergeometric test, (*) *P* <0.05.

**Figure 5:**
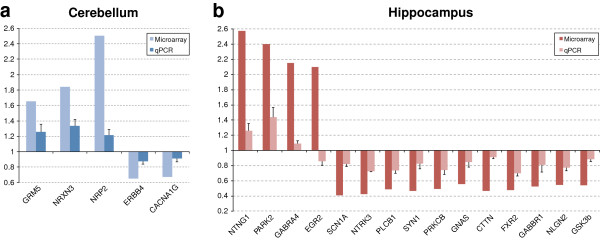
**Quantitative PCR validation of differentially expressed genes.** A selected number of differentially expressed genes in the *En2*^*-/-*^ cerebellum **(a)** and hippocampus **(b)** were validated by qPCR. Relative mRNA expression level (fold expression) as obtained by qPCR performed on whole cerebellum or hippocampus extracts of adult wild type (WT) and *En2*^*-/-*^ mice. Correlation of fold expression from qPCR (light color bars) and microarray (dark color bars) results was calculated using Pearson’s Correlation. Except for *Egr2*, qPCR results for all the evaluated genes showed significant correlation with microarray results (R = 0.84; *P* <0.05). Values are expressed as each gene/*L41* comparative quantitation ratios normalized on the expression of WT (mean ± s.e.m of three replicates from pools of four animals per genotype; *P* <0.01, Student’s *t*-test, WT versus *En2*^*-/-*^.

To validate microarray findings with qPCR analysis, we selected eight and eighteen representative genes from the cerebellum and the hippocampus differentially expressed gene lists, respectively. The selected genes reported differential expression values in microarray experiments ranging between 0.41 fold decrease to 2.5 fold increase. With qPCR, five of the eight selected genes showed statistically significant differential expression in the *En2*^*-/-*^ cerebellum [glutamate receptor, metabotropic 5 (*Grm5*) *P* = 0.031; neurexin III (*Nrxn3*) *P* = 0.032; neuropilin 2 (*Nrp2*) *P* = 0.009; v-erb-a erythroblastic leukemia viral oncogene homolog 4 (*Erbb4*) *P* = 0.003; calcium channel, voltage-dependent, T type, alpha 1G subunit (*Cacna1g*) *P* = 0.003] (Figure 5a). Fifteen genes of the eighteen selected were instead significantly changed in the *En2*^*-/-*^ hippocampus [netrin G1 (*Ntng1*) *P* = 0.008; Parkinson disease (autosomal recessive, juvenile) 2, parkin, (*Park2*) *P* = 0.004; gamma-aminobutyric acid (GABA) A receptor, subunit alpha 4 (*Gabra4*) *P* = 0.007; early growth response 2 (*Egr2*) *P* = 0.007; sodium channel, voltage-gated, type I, alpha (*Scn1a*) *P* = 0.001; neurotrophic tyrosine kinase, receptor, type 3 (*Ntrk3*) *P* = 0.0003; phospholipase C, beta 1 (*Plcb1*) *P* = 0.0001; cortactin (*Cttn*) *P* = 0.0005; synapsin I (*Syn1*) *P* = 0.007; fragile X mental retardation, autosomal homolog 2 (*Fxr2*) *P* = 0.0004; protein kinase C, beta (*Prkcb*) *P* = 0.001; gamma-aminobutyric acid (GABA) B receptor, 1, (*Gabbr1*) *P* = 0.03; neuroligin 2 (*Nlgn2*) *P* = 0.001; glycogen synthase kinase 3 beta (*Gsk3b*) *P* = 0.003; GNAS (guanine nucleotide binding protein, alpha stimulating) complex locus (*Gnas*) *P* = 0.01] (Figure 5b). Except for *Egr2,* in all tested genes the expression differences reported by qPCR correlated with the microarray data (Pearson r = 0.84, *P* < 0.00005).

## Discussion

To date, more than 500 autism-associated genes have been identified (SFARI Gene; gene.sfari.org; updated mar/2013); yet the etiology of ASD remains essentially unknown [6,7]. The significance of animal models in ASD research has been widely recognized as important for unraveling the molecular, cellular, anatomical, electrophysiological and behavioral consequences of gene dysfunction in ASD. Here, we present a transcriptome analysis in a mouse model of ASD of two brain areas, the cerebellum and the hippocampus, areas that are profoundly affected in ASD patients. Despite the small number of samples used for the microarray analysis and the sample gender heterogeneity, the low genetic variance among individuals allowed a reasonable statistical power for our bioinformatic analysis. Our study revealed that the molecular signature of these two brain regions shares convergent pathological pathways with ASD, including abnormal synaptic transmission and increased immune response. Furthermore, when directly compared to the repository of the SFARI database (gene.sfari.org), our differentially expressed genes in the hippocampus show an enrichment of ASD-associated genes significantly higher than previously reported [41].

Transcriptome analysis has been employed to unravel common pathways based on the assumption that the core phenotypes of ASD may be caused by convergent molecular mechanisms [6]. Several studies have analyzed genome-wide expression profiles of lymphoblastoid cell lines and blood samples from ASD patients, pointing to an upregulation of immune genes as key mechanisms in the pathogenesis of ASD [8]. Despite the limited source of brain tissue samples from ASD cases and the technical restrictions, studies of ASD brain transcriptome are emerging as strategic for uncovering functionally relevant alterations in gene expression. A previous microarray study found alterations of glutamatergic neurotransmission in ASD cerebellum [39], and expression profiles from ASD patient temporal cortices showed upregulation of genes involved in innate immune response and downregulation of several neurodevelopmental genes [40]. Moreover, the transcriptome profiles from three different brain regions (frontal cortex, temporal cortex and cerebellum) of nineteen autism cases and seventeen controls were investigated recently using classical differential expression analysis and a network-based approach [9]. These analyses showed upregulation of genes involved in immune response and downregulation of genes involved in synaptic function and vesicular transport [9]. Our results are in accordance with these findings. Using gene ontology enrichment, integrated gene-network analysis and mouse phenotypes analysis, we report significantly enriched functions and pathways that were previously associated to ASD [42]. In detail, we found increased immune response and major histocompatibility complex-related immunity in the *En2*^*-/-*^ cerebellum; decreased and abnormal neurotransmission and increased seizures in the *En2*^*-/-*^ hippocampus [see Additional file 5 for details]. Moreover, by direct comparison with the SFARI repository of ASD-related genes, we show that the gene expression changes observed in the *En2*^*-/-*^ hippocampus were significantly enriched in ASD-related genes. Furthermore, the proportion of ASD-associated genes enrichment in *En2*^*-/-*^ hippocampus was significantly higher than previous studies (Table 2) when compared with Voineagu *et al.*[9], likely the most comprehensive transcriptome study of ASD post-mortem brain to date. In the case of the cerebellum, in contrast to Voineagu *et al.*[9] we did not find significant enrichment of ASD-associated genes in *En2*^*-/-*^ mice. Such difference could be the result of the complex structural and cytoarchitectural abnormalities in *En2*^*-/-*^ cerebellum [20,21] and the consequent phenotypical variability, or could simply reflect differences between mouse and human phenotypes, as the incidence of cerebellar hypoplasia was not reported in the diagnostic criteria used in the study [9]. Remarkably, *EN2* was among the differentially expressed genes found in Voineagu *et al.*[9], confirming our evidence about the role of *En2* in the neuropathology of ASD, and in anterior brain structures [27].

Among the differentially expressed genes, *Grm5, Nrxn3* and *Scn1a* are of particular interest for ASD. *Grm5* encodes mGluR5, a G-protein coupled receptor for the neurotransmitter glutamate [43]. In a recent study, mGluR5 has been shown to participate in the pathogenesis of fragile X syndrome (FXS) while genetic downregulation of *Grm5* was able to compensate for some of the symptoms in a mouse model of FXS [44]. Furthermore, *Grm5* was shown to be downregulated in hippocampal neurons lacking *Shank3*, another ASD-associated gene [45]. These data support a central role for *Grm5* in neurobiological pathways related to ASD pathogenesis. Our results show an increased expression of *Grm5* in the cerebellum of *En2*^*-/-*^ mice, suggesting a role of *Grm5* in the cerebellar phenotype of these mice*.* The contribution of *Grm5* and its interaction with *Fmr1* in the *En2*^*-/-*^ hippocampus remains to be established and could open new perspective of pharmacological and genetic rescue of the ASD-related phenotype of these mice.

*Nrxn3* encodes neuronal adhesion proteins of the Neurexin (NRXN) family. NRXNs are presynaptic cell adhesion proteins that form trans-synaptic complexes with their postsynaptic counterpart neuroligins (NLGNs) and have important roles in synapse development and function [46]. Recently, a report of hemizygous and *de novo* deletions involving *NRXN3* in ASD families provided strong support for a causative link between the loss of *NRXN3* and the development of ASD [47]. Our results of an increased expression of *Nrxn3* in the cerebellum suggest alterations in Purkinje cell synaptic formation, where NRXNs have been shown to participate to the formation of glutamatergic synapses through interaction with Cerebellin 1 precursor protein (also downregulated in the *En2*^*-/-*^ cerebellum) and GluR∂2 [48,49].

*Scn1a* encodes the voltage-gated sodium channel alpha subunit. *De novo* null mutations in *SCN1A* result in severe myoclonic epilepsy of infancy [50]. *SCN1A* mutations have been associated to a number of neurological disorders, including generalized epilepsy with febrile seizures plus, Dravet syndrome, borderline myoclonic epilepsy in infancy, intractable childhood epilepsy with generalized tonic-clonic seizures, familial hemiplegic migraine, and a number of cryptogenic focal and generalized epilepsies. Recently, *de novo* mutations in *SCN1A* have been associated with ASD [51], and a report of a recognized mutation in *SCN1A* suggests a wide phenotypic variation of the gene mutations causing a variety of neurologic disorders, including ASD [52]. In mice, heterozygous loss-of-function mutation in *Scn1a* (*Scn1a*^*+/-*^), reproduces several of the symptoms associated to the human mutation, such as thermally induced and spontaneous seizures, premature death, ataxia and sleep disorder [53,54]. *Scn1a*^*+/-*^ mice show both cognitive deficits and autistic traits that are caused by impaired GABAergic neurotransmission and can be rescued by drug treatment. *Scn1a* down-regulation in the *En2*^*-/-*^ hippocampus could contribute to the abnormal excitability and altered GABAergic neurotransmission shown in these mice by our previous studies [27,30]. Pharmacological rescue of the hippocampal phenotype in the *En2*^*-/-*^ with GABAergic drugs is currently under investigation.

Anomalies in the cerebellum are the most reproducible neuropathological alterations in ASD patients. Several cerebellar abnormalities have been observed in mouse models of both *En2* gain- and loss-of-function. Ectopic overexpression of *En2* in Purkinje cells during late embryonic and postnatal cerebellar development results in reduced cerebellar volume and loss of Purkinje cells and other cerebellar neurons [55,56]. Interestingly, *En2* knock-out causes defective cerebellar patterning, reduced Purkinje cell number and abnormal dendritic foliation [10,57], indicating that alterations in *En2* expression levels during development cause similar phenotypes. Furthermore, deficits in social behaviors as well as defective spatial learning and memory were also reported in *En2*^*-/-*^ mice [24-26]. A recent epigenetic analysis of *EN2* promoter methylation in the cerebellum of ASD individuals indicated hypermethylation of the promoter region and persistent upregulation of the gene. The authors report that promoter hypermethylation is normally associated with a decrease in gene expression and suggest the possibility of a developmental mechanism intended to support downregulation of *EN2* during perinatal development [19]. Taken together, this evidence suggests that an overall imbalance in *EN2* expression may be relevant for ASD pathogenesis, as it could produce alterations in critical brain functions. Comparable evidence of a similar dosage effect has been reported in the case of mutations of other genes critically involved in gene expression regulation and maintenance of synaptic and neuronal homeostasis, such as *MECP2* and *ARX*[58,59]. It remains to be established whether *En2* overexpressing mice display abnormal behaviors relevant to autism. Microarray data have been produced for *En2* overexpressing Purkinje cells on a different platform; however, the results overlap only marginally with the herein reported study [60].

## Conclusions

Using transcriptome analysis, we identified over 800 genes differentially expressed in the cerebellum and hippocampus of *En2*^*-/-*^ mice. Despite the small number of samples used and the relatively small statistical power, our study is the first to analyze molecular changes occurring in two brain structures with neuroanatomical alterations relevant to ASD in a mouse model of this disease. Our bioinformatic analysis of the molecular signature of *En2*^*-/-*^ cerebellum and hippocampus shows a significant convergence of neurobiological pathways previously linked to ASD pathology in brain samples from ASD patients. Overall, the present study points to a strong impact of transcriptome analysis on mouse models for identifying neurobiological pathways commonly altered when ASD genes are disrupted in a human patient and in a mouse model alike. Furthermore, together with the frequent association of cerebellar neuroanatomical alterations to the neuropathology of ASD, our molecular analysis suggests a contribution also for the hippocampus, where molecular changes relevant to ASD may occur also in human patients. This notion is supported by the consistent enrichment of ASD-related genes in the *En2*^*-/-*^ hippocampus compared to the cerebellum and to other similar studies performed on ASD patient tissue samples [41]. Finally, our results confirm the *En2*^*-/-*^ mouse model of ASD as a valuable tool for investigating neuroanatomical, behavioral, as well as molecular alterations related to ASD.

## Availability of supporting data

The data sets supporting the results of this article are available in the GEO repository, GSE51612.

## Abbreviations

ASD: Autism spectrum disorders; pfp: Percentage of false positives; DAVID: Database for Annotation, Visualization and Integrated Discovery; FXS: Fragile X syndrome; GABA: Gamma aminobutyric acid; GO: Gene ontology; IPA: Ingenuity pathway analysis; MHC: Major histocompatibility complex; MPO: Mammalian phenotype ontology; NLGN: Neuroligin; NRXN: Neurexin; RP: Rank product; SFARI: Simons Foundation and Autism Research Initiative; SNP: Single-nucleotide polymorphism; WT: Wild type.

## Competing interests

The authors declare that they have no competing interests.

## Authors’ contribution

PS, GP and YB conceived the study. PS, GP, VA, GZ and SG performed microarray and qPCR experiments. PS, GP and ED performed bioinformatic analysis. PS, GP and YB analyzed data. PS and YB wrote the paper. PS, SC and YB provided funding. All authors read and approved the final manuscript.

## Supplementary Material

Additional file 1 RT-qPCR primers used in the study. Click here for file

Additional file 2 **Differentially expressed gene in the cerebellum and in the hippocampus of *En2***^***-/-***^** mice.** Tables showing differentially expressed genes in the *En2*^*-/-*^ (**a**) cerebellum and (**b**) hippocampus with fold change expression compared to WT littermates, differential expression *P* value and percentage of false prediction (pfp) calculated with RankProd. *P* value cut-off *P* <0.01 for the cerebellum and *P* <0.005 for the hippocampus. Click here for file

Additional file 3 **Genes differentially expressed in both *En2***^***-/-***^**cerebellum and hippocampus.** Genes commonly regulated in the *En2*^*-/-*^ cerebellum and hippocampus with differential expression *P* value and fold change. Click here for file

Additional file 4 ***En2 *** **full-length expression.** Quantitative PCR analysis of En2 full-length expression in the WT and *En2*^*-/-*^ cerebellum and hippocampus. Values are expressed as *En2*/*L41* comparative quantitation ratios normalized on the expression of WT in the cerebellum (mean ± s.e.m of three replicates from pools of three animals per genotype; *P* <0.01, Student’s *t*-test, WT versus *En2*^*-/-*^). Click here for file

Additional file 5 **Integrated gene-network analysis and gene ontology enrichment.** Tables a-f show the ten most significant disease and functional annotations obtained with Ingenuity Pathways Analysis (IPA) for *En2*^*-/-*^ cerebellum (a-c) and *En2*^*-/-*^ hippocampus (d-f) differentially expressed genes. Functional categories and annotations are shown with their *P* value, the predicted activation state, the activation z-score, the associated genes and the number of enriched genes. Activation z-score >2 or < -2 indicates significantly increased or decreased annotations. Tables g-j show gene ontology analysis details for *En2*^*-/-*^ cerebellum (g, h) and *En2*^*-/-*^ hippocampus (i, j) differentially expressed genes. Gene ontology annotations are shown with gene counts and corrected *P* values. Significant disease and functions for up- (c, f, h) and downregulated (b, e, j) genes are shown separately. Click here for file

Additional file 6 **Tissue expression of differentially expressed genes in *En2-/- *** **hippocampus.** Table showing gene ontology tissue expression annotations for differentially expressed genes in the *En2*^*-/-*^ hippocampus. No significant tissue expression annotations were found for differentially expressed genes in *En2*^*-/-*^ cerebellum. Click here for file
