# Supplementary figures and images for: Transcriptome profiling in engrailed-2 mutant mice reveals common molecular pathways associated with autism spectrum disorders
Source: Mol Autism. 2013 Dec 19;4:51. doi: 10.1186/2040-2392-4-51 (PMC3896729; doi:10.1186/2040-2392-4-51)

# En2 expression

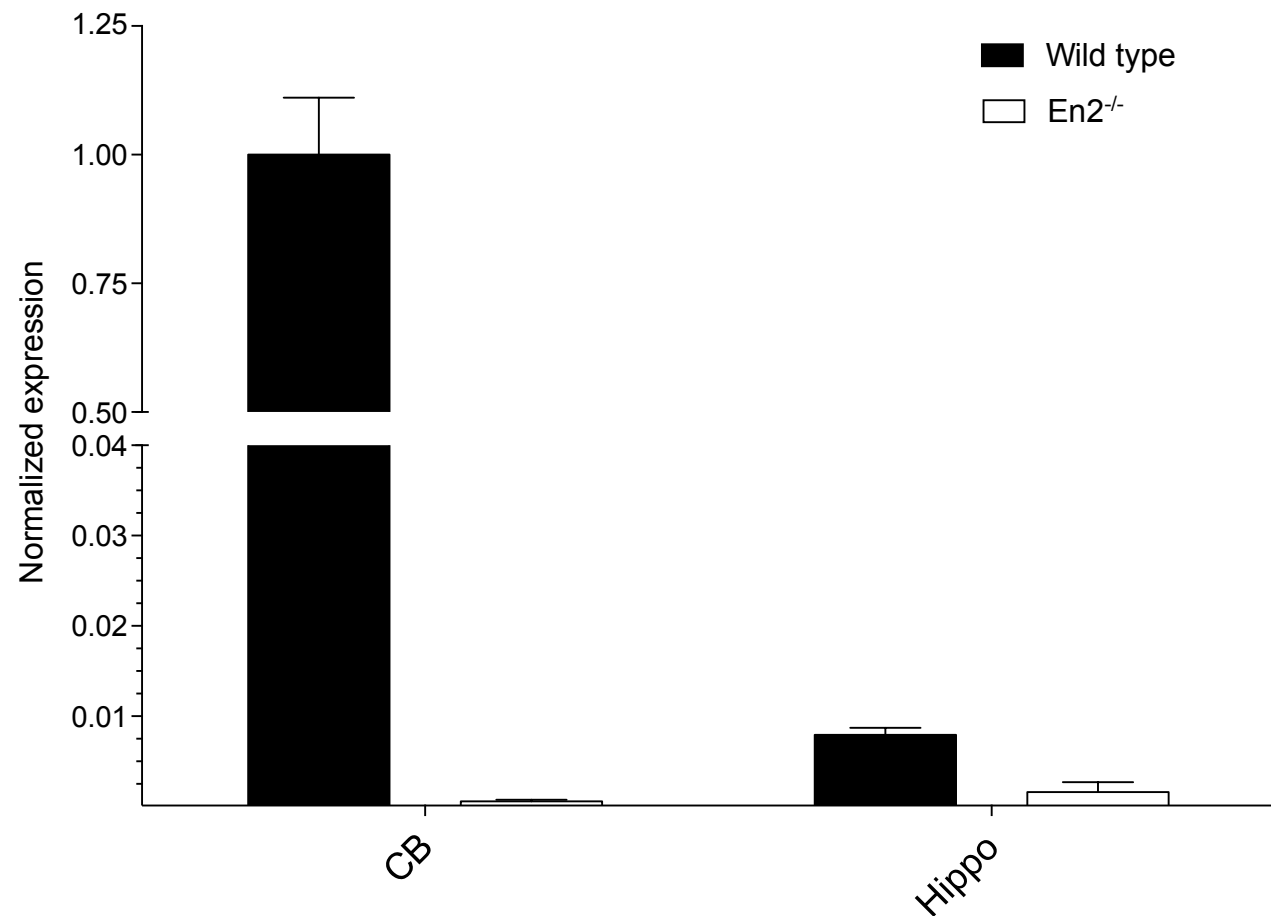

Supplement: Additional file 4 — En2 full-length expression. Quantitative PCR analysis of En2 full-length expression in the WT and En2 -/- cerebellum and hippocampus. Values are expressed as En2/L41 comparative quantitation ratios normalized on the expression of WT in the cerebellum (mean ± s.e.m of three replicates from pools of three animals per genotype; P <0.01, Student’s t-test, WT versus En2 -/- ). [file 2040-2392-4-51-S4.pdf]
